# Acne treatment challenges – Recommendations of Latin American expert consensus^⋆^

**DOI:** 10.1016/j.abd.2023.09.001

**Published:** 2024-02-23

**Authors:** Marco Rocha, Franz Barnes, Jemena Calderón, Leonel Fierro-Arias, Carlos Eduardo Montealegre Gomez, Carla Munoz, Obregón Jannell, Patricia Troieli

**Affiliations:** a Universidade Federal de São Paulo, São Paulo, SP, Brazil; b Sociedade Brasileira de Dermatologia, São Paulo, SP, Brazil; c Iberolatin-American College of Dermatology (CILAD), Panamá City, Panama; d Sociedad Ecuatoriana de Dermatologia, Ecuador; e Hospital General de Mexico and American British Cowdray (ABC) Medical Center, Mexico City, Mexico; f Mexican Society of Dermatologic & Oncologic Surgery, Iberolatin-American College of Dermatology (CILAD), Mexico City, Mexico; g Universidad de Antioquia, Medellin, Colombia; h Asociación Colombiana de Dermatología y Cirugía Dermatológica, Medellin, Colombia; i Clinica Santa Maria, Santiago de Chile, Chile; j Sociedad Chilena Dermatología and Iberolatin-American College of Dermatology (CILAD), Santiago de Chile, Chile; k Lima Dermatology Center Clinic and Esthetic, Lima, Peru; l Sociedad Peruana de Dermatologia, Lima, Peru; m School of Medicine University of Buenos Aires, Buenos Aires, Argentina; n Argentine Dermatology Society, Iberolatin-American College of Dermatology (CILAD), American Academy of Dermatology and European Academy of Dermatology, Buenos Aires, Argentina

**Keywords:** Acne, Isotretinoin, Thiamidol, Latin America, Hyperpigmentation

## Abstract

**Background:**

Acne is a chronic inflammatory disorder of the pilosebaceous unit that is associated with a negative impact on quality of life, causing anxiety, depression, and poor self-esteem. The treatment of acne is not simple and presents some new challenges. This article addresses important issues faced by dermatologists on their daily, some of them specific for Latin America.

**Objective:**

To discuss daily practice recommendations when managing acne patients.

**Methods:**

A literature review was conducted by a group of eight experts with extensive experience in the field of acne. The results of the data review were presented at an initial kick-off meeting to align the consensus topics. Two e-surveys using the Delphi methodology and an interim group webinar meeting were held.

**Results:**

The expert panel reached a consensus on all proposed key statements, providing scientific support to help dermatologists and healthcare providers make acne management decisions on topics that can be challenging in the everyday practice of dermatology, such as the characteristics of Generation Z or the importance of the maintenance phase of adult acne treatment.

**Conclusion:**

This article provides current recommendations for managing acne patients. The high level of agreement achieved based on the latest evidence supports the best acne therapeutic choices in both established topics and new important issues that have emerged in recent years, such as the impact of social media, Generation Z characteristics, and transgender male patient specifics.

## Introduction

Acne is a chronic immune-inflammatory disorder of the pilosebaceous unit (ED80-ED80.Z according to the WHO-International Classification of Disorders ICD-11 2022).^1^ It is clinically characterized by comedones, papules, pustules, nodules, or cysts. It can often cause sequelae such as scarring and dyschromia.

Having acne has been associated with a negative impact on quality of life, leading to anxiety, depression, and reduced self-esteem.^2^

The disorder affects more than 80%‒85% of adolescents and young adults,^3^ but sometimes remains a significant problem into adulthood, especially in women. More than 40% of women are affected by acne after the age of 25.^4^

It must be understood that this epidemiological behavior should not differ from what happens in Latin America,^5^ where acne is the most common dermatologic disorder.^6^ However, some differences could be observed due to demographic, racial/ethnic, and exposome influences, among others, specific to the region.^7^

Understanding the specific challenges of the Latino population, as analyzed by professionals working in the region, is crucial for improving treatment outcomes. Through these discussions, healthcare professionals can gain valuable insights into the unique needs of Latino patients, enabling them to develop tailored treatment approaches that effectively address their concerns. In this article, only experienced dermatologists from different countries in Latin America sought to identify and discuss the main barriers related to acne and how to change them.

## Methodology

### Expert panel

Professionals with extensive experience in the management of acne were identified in their respective countries as individuals who work in academic settings, deliver lectures at conferences, and possess in-depth knowledge in providing care for patients with acne. The expert panel comprised eight dermatologists from Latin America, representing countries such as Argentina, Brazil, Chile, Colombia, Peru, Ecuador, Mexico, and Panama.

The process was led by the chairman, who was also part of the panel and responsible for the design of the Delphi survey.

### Delphi process modified

**Figure 1:**
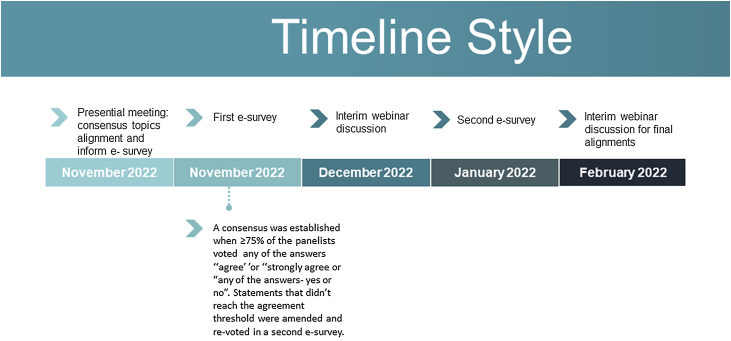
Delphi process modified for Acne Consensus.

The Delphi modified methodology^8^ consisted of reviewing the literature to update the most relevant topics in the clinical management of acne and potential gaps, as well as to identify the specificities of acne in the Latin American population. Searches were performed using PubMed Central, Cochrane, and Lilacs databases from January 2000 to December 2022, including English and Spanish articles. These data were presented by the expert panel during a face-to-face meeting to guide the discussion. This was followed by two e-surveys and a live virtual meeting between the first and second e-surveys (Fig. 1). The results of the e-surveys were presented by the chairman.

The e-surveys were designed to assess the level of agreement with each statement. Two different ways of approaching the questions were used in the first e-survey. Sixteen questions measured the answer according to an answer range: “strongly disagree”, “disagree”, “agree”, “strongly agree”, or “unable to answer”. A consensus was reached when ≥75% of panelists voted “agree” or “strongly agree”. For eleven questions, the answer was measured as “yes” or “no” and a written explanation for the answer was selected. A consensus was reached when ≥ 75% of panelists voted for one of the answers. The explanations were analyzed by the chairman and relevant issues were presented for discussion during the first interim webinar.

Two topics in the e-survey didn't reach the minimum 75% agreement level. These topics were discussed during the interim web meeting, a new literature review was carried out and a new vote was taken.

The e-surveys were programmed and managed by Sprim Brazil to ensure blinding.

In the first round, sixteen out of seventeen questions reached a consensus with ≥75% of votes “agree” or “strongly agree” (Table 1). The result of the voting on the consensus statement is given in parentheses (e.g., 7/8 voted “agree” or “strongly agree”) after each topic discussed.

**Table 1:** Statements with consensus during the first e-survey with ≥75% of “agree”, “strongly agree” votes.

**1 - Quality of life**	100%
Acne is a condition that significantly affects quality of life. The tools available to assess its impact **are impractical for daily use** in the office and take a long time to apply and analyze.	
**2 - Social Media**	100%
Previous research analyzing the main social media sites (Instagram®, Facebook®, Youtube® and TikTok®) proved that acne and its treatment is one of the most frequently searched medical topics, but most content producers are not dermatologists. Then, **it is often necessary to deconstruct concepts that have been wrongly absorbed in order to restart an effective treatment.**	
**3 - Medication Use Investigation**	100%
In order to **find negative factors that may affect the effectiveness of acne treatment**, active questioning about the use of manipulated compounds, topical and systemic medications, vitamins, hormonal implants, and contraceptive methods should always be performed.	
**4 - Red flag: acne in children <7 years old**	100%
The presence of inflammatory acne in children between 1 and 7 years of age is not common. **The patient should always be referred for endocrinologic evaluation to rule out precocious puberty.**	
**5 - Skincare I – Dermocosmetics**	100%
Specific dermocosmetic use for treating acne is a feasible tool. In mild cases, it can be used alone and in more severe cases it can be associated with the treatment protocol proposed by the guidelines.	
**6 - Skincare II**	100%
Mild cleansers and related non-comedogenic moisturizers are recommended for **better tolerance** of topical medications, increasing treatment success.	
**7 - Acne treatment ‒ Women's topical treatment >25 years old**	87.5%
There is an increasing number of acne cases in women over the age of 25 years of age. The cause is not yet fully elucidated. When starting the treatment of these patients, it is important to consider that their skin is more sensitive due to the presence of acne concomitant with aging process.	
**8 - Acne treatment – Isotretinoin I**	100%
Currently, when oral isotretinoin is required, **treatment should be based on clinical evaluation rather than cumulative dose alone.** If necessary, the treatment should be continued, even after 150 mg/kg.	
**9 - Acne treatment – Isotretinoin II**	87.5%
After the use of oral isotretinoin, **a one-year maintenance topical treatment** should be recommended for all patients in order to reduce relapse rates.	
**10 - Acne treatment ‒ Isotretinoin** in transgender men	87.5%
Frequent laboratory testing is important with oral isotretinoin use in transgender men, as chronic testosterone use may also be associated with dyslipidemia and hepatotoxicity.	
**11 - Diet**	100%
Although more research is needed on diet and acne, **it is important to provide the most current information on the subject**, such as the negative relationship with foods with a high glycemic index/load, dairy products including whey protein, and the need to eat more fruits and vegetables.	
**12 - Probiotics**	100%
Although there are some studies evaluating the use of oral probiotics in the treatment of acne, their prescription is still not part of the routine for patients **until better evidence is available**.	
**13 - Scars I**	100%
Family history of acne scarring, as well as the intensity of the disorder, time without treatment, and manipulation of the lesions, influence the presence of atrophic acne scarring. These factors should be discussed with the patient prior to treatment.	
**14 - Scars II**	100%
All acne cases, even mild, must be treated effectively as scars can appear after any acne intensity.	
**15 - Hyperpigmentation**	100%
Acne-induced hyperpigmentation is a common problem. Its treatment is challenging, and previous studies have associated its intensity with the degree of acne, phototype, and duration of the inflammatory process. Due to migratory factors, the large presence of mestizos, and the geographic location of most Latin American countries, **most patients have acne-induced hyperpigmentation** and desire a rapid improvement.	
**16 - Dermatologic procedures**	100%
Dermatologic procedures can be associated with the treatment of acne and pigmentation, such as peels and lasers, **but they are not mandatory and significantly increase the cost to the patients.**	

Ten out of eleven questions, measured by a “yes” or “no” answer, achieved consensus with ≥75% “yes” answers (Table 2).

**Table 2:** Consensus statements in the first e-survey with ≥75% “yes” responses.

**Social Media ‒ medical presence**	100%
Having a dermatologist talking about acne on social media is important.	
**Generation Z**	100%
Generation Z are digital natives, with a huge presence on social media, with a strong social and environmental responsibility, and are used to having quick answers while also having high rates of anxiety and depression. A specific language, associated with the use of digital resources (WhatsApp® messages/direct message-Instagram®) is important to connect with Generation Z, favoring the follow-up of physical, psychological symptoms and the treatment.	
**Transgender man**	100%
Well-conducted studies to guide acne treatment in male transgender patients are still lacking.	
**Adult female acne ‒ systemic treatment**	75%
When it is necessary to use systemic medications for the treatment of acne, it is preferable to start with spironolactone, since these patients have a chronic clinical condition and, in their history, they have often used systemic antibiotics.	
**Adult female acne ‒ maintenance treatment**	100%
Maintenance treatment should always be indicated for these patients.	
**Makeup**	100%
Non-comedogenic makeup may be recommended for acne patients to reduce the impact of the presence of lesions early in treatment.	
**Dermocosmetics**	100%
Whitening dermocosmetics can be combined with acne treatment to accelerate the improvement of macular hyperpigmentation concomitantly with the improvement of acne.	
**Intermittent Dose**^a^ (isotretinoin)	87.5%
In accordance with the studies that have already been published, it is preferable to avoid the regimen of intermittent doses of oral isotretinoin.	
**Informed Consent related to isotretinoin**	100%
It is important that a medication as effective as oral isotretinoin continues to be safely prescribed with consent and only by physicians experienced in monitoring it (dermatologists).	

a Intermittent dose means take the medication for a few days with intervals of days/weeks between administrations.

During the second e-survey, two topics that did not reach consensus in the first e-survey were reassessed. In the second e-survey, consensus was achieved on these 2 topics with 100% “agree” answers and ≥75% “yes” answers in a “yes/no” question (Table 3, Table 4).

**Table 3:** Consensus statement during the second e-survey with 100% “agree” votes.

**Pediatric acne**	100%
When present in younger children, the same topical medications are indicated as for children over 9 years of age.

**Table 4:** Consensus statement in the second e-survey with ≥75% “yes” answers.

**Adult female acne**	75%
Patients with persistent acne, whether or not accompanied by seborrhea, without other signs and symptoms of hyperandrogenism do not require hormone evaluation testing.	

## Results and discussion

### Quality of life

Acne is a condition that has a significant negative impact on quality of life.^9^ In addition, long-term sequelae, such as the presence of scarring after acne has resolved, cause significant emotional, social, and functional concerns. Familiarity with the psychological aspects of acne is important for holistic patient management. Psychological factors can exacerbate acne and decrease patient compliance.^3^

Often, the patient's perception of the disorder may differ from the dermatologist's assessment, and the intensity of the disorder is not always directly related to the degree of psychological impact. There are several psychometric tools to assess this impact, among them Acne-QoL and Dermatology Life Quality Index (DLQI). Some of them have been translated and published in Portuguese and Spanish. However, in day-to-day practice, these tools are impractical and take a long time to apply and analyze (8/8 -100% of agrement). There is a lack of a quick and practical tool to assess and monitor the psychological impact on acne patients.

### Social media

Social networks have become a means of sharing information about health-related issues.^10^ Currently, all of our patients are increasingly exposed to controversial content on various social media platforms, which is emerging as a public health problem that may, in the long run, prevent us from providing fully effective care and delay effective treatment.

To improve this situation, the authorss suggest developing educational campaigns to help users reject false or misleading information while promoting the dissemination of rigorous, evidence-based dermatology knowledge.

Dermatologists should be encouraged to increase their presence on various social media platforms, always considering privacy and ethical principles of their position as physicians, and to build a more solid base of correct orientations within these channels^11^ (8/8).

### Generation Z

Generation Z individuals were born between 1995–2010, so they are the first generation to be born with full access to the Internet (“digital natives”). Several studies have looked at how to approach education with this generation and how to use social media as an educational tool. A 2020 study of nursing students^12^ found that participants used social media for an average of 1.37 hours for clinical learning and about twice as long for personal purposes. Therefore, using social networking to connect, engage, and educate this generation of patients is a useful and necessary tool.^12^, ^13^, ^14^, ^15^

Therefore, this expert panel believes that a specific language associated with the use of digital resources (WhatsApp® messages/direct messages on Instagram®) is important to connect with Generation Z, favoring the follow-up of physical and psychological symptoms and the treatment of these patients (8/8).

### Transgender male patient

Transgender individuals do not identify as the biological sex assigned at birth, and some seek medical assistance for interventions aimed at phenotypically restoring their gender identity. To maintain levels at physiological levels, between 320‒1000 ng/dL, transgender men must use testosterone continuously.^16^

Today, there is still a lack of well-conducted studies to guide the treatment of acne in transgender male patients (8/8). There is also very little scientific literature that analyzes the problems induced by hormone therapies in transgender male patients in the short and long term. A retrospective study conducted by the Mayo Clinic (USA) from 1986 to 2018 analyzed 222 transgender people, 36% of whom were female-to-male, and 91.4% of whom were receiving hormone therapy. Acne was the most commonly diagnosed side effect, present in 78.7% with an average onset of 11.5 months of testosterone therapy.^17^

The psychosocial impact of acne may exacerbate gender dysphoria and the already high rates of psychiatric comorbidity in transgender patients.^18^

The number of reported cases of severe or resistant acne in these patients responding only to oral isotretinoin is increasing, although it has been suggested that dermatologic changes are unrelated to plasma levels of androgens.^19^, ^20^, ^21^, ^22^

It is important to note that these patients can still become pregnant. Therefore, contraception should always be used. The interaction between chronic testosterone uses and isotretinoin has not been widely studied, so this study group suggests frequent laboratory follow-up in these cases, especially analyzing liver function, lipids, and pregnancy tests (7/8).^23^

### Pediatric acne ‒ when to alert

Childhood acne refers to lesions that are suggestive of acne in patients between the ages of 1 and 7 years of age. It is a very rare presentation, and a complete and thorough clinical evaluation must always be performed. This group considers that any patient with inflammatory acne in this age group should be referred to endocrinology to actively rule out other pathologies (8/8).^24^

### Adult female acne (AFA)

#### Topical treatment

It is important to consider that their skin is more sensitive due to the presence of acne and also due to skin aging when starting treatment of these patients (7/8). Topical therapies alone are usually sufficient for the successful treatment of mild cases.^25^ A combination of topical and systemic treatment is required for most moderate and severe cases.^25^

Table 5 describes in detail the main topical substances (retinoids,^26^ azelaic acid,^27^, ^28^, ^29^ topical antibiotics,^30^ benzoyl peroxide,^31^ dapsone,^32^ and fixed-combinations)^33^ most used for these cases.

**Table 5:** Topical treatments available for adult female acne (AFA).

Substance	Recommendation
**Retinoids**	Third-generation topical retinoids (adapalene 0.1%) are recommended for the treatment of mild comedonal acne and mild-to-moderate inflammatory acne.^26^
**Azelaic acid**	Azelaic acid 15% gel is recommended as a first-line monotherapy for both non-inflammatory and inflammatory acne in adult women.^27^ Azelaic acid is also indicated for the treatment of post-inflammatory hyperpigmentation.^28^ This option is safe for use during pregnancy and breastfeeding.^29^
**Topical antibiotics**	Its isolated use should not be prescribed.^30^
**Benzoyl peroxide**	BPO is recommended as monotherapy for mild-to-moderate acne in AFA, in low concentrations.^31^
**Dapsone**	Dapsone 5% gel shows only modest to moderate efficacy in reducing inflammatory acne lesions.^32^
**Fixed combination**	Topical combination therapy, retinoid-BPO, antibiotic-BPO and antibiotic-retinoid, is recommended for inflammatory acne in adult females.^33^

BPO, Benzoyl Peroxide.

#### Systemic treatment

In most cases, AFA patients have mild to moderate severity, with few comedones, persistent disorder, and no hormonal changes. In more severe cases, systemic therapy may be required. The use of oral antibiotics, preferably with tetracycline, is indicated by guidelines, but relapse rates are very high.^34^

According to this group, if systemic therapy must be used to treat adult acne, starting with spironolactone may be preferable because of these patients' chronic clinical condition and history of frequent use of systemic antibiotics (6/8).

Although spironolactone is off-label for the treatment of acne, it is highly effective. However, it is used much less frequently than oral antibiotics. The recommended doses for starting treatment should be around 100 mg daily, preferably divided into two doses, after meals. The drug has a short half-life of 6 to 9 hours and depends on food for perfect absorption.^35^

Co-administration of diuretics and tetracyclines may result in decreased renal function, manifested by increases in serum creatinine and blood urea nitrogen, and should be accompanied by laboratory analysis.^36^

#### Maintenance treatment

The usual evolution of AFA occurs with frequent relapses, making maintenance treatment essential.^37^ Topical retinoids such as adapalene 0.1% or azelaic acid 15% gel are recommended as first-line maintenance therapy for adult women.^27^

New options have emerged, such as clascoterone, but there are still no studies for the maintenance phase. In some cases, the use of low doses of spironolactone or special regimens of low doses of oral isotretinoin may be necessary.^38^

### Skincare

This group recommends that the use of a skincare routine may be an important strategy in general and specifically in the treatment of acne in terms of patient satisfaction and adherence. Appropriate dermocosmetic recommendations are necessary to reduce side effects and improve treatment efficacy. This includes advice on the correct sunscreen, cleanser and moisturizer, with or without anti-acne ingredients, and make-up (8/8).

Almost all patients on systemic treatment, especially those using isotretinoin or topical retinoids and benzoyl peroxide, experience varying degrees of side effects such as irritation, burning, dry skin, erythema, and scaling, leading to poor adherence and compromising treatment efficacy. The overall risk of poor adherence to acne treatment is approximately 50% to 60%.^39^

The authors recommend the use of mild cleansers (syndets) and associated non-comedogenic moisturizers to improve the tolerability of topical medications and increase the chances of treatment success.^40^

Moisturizers and products containing sunscreen with a Sun Protection Factor (SPF) of at least 30 are important components of the acne patient's management to complement the pharmacologic regimen.

Acne patients should be advised to avoid oil-based and comedogenic sunscreens whenever possible, as these products may aggravate their acne vulgaris.

Dermocosmetics with whitening action can be combined with acne treatment to accelerate the improvement of post-inflammatory hyperpigmentation along with the improvement of acne (8/8).

Table 6, Table 7 list key ingredients for the proper selection of moisturizers, gentle cleansers, and Dermocosmetics (DCs).

**Table 6:** Moisturizer and cleanser.

Moisturizers (components)	Gentle cleansers (on acne-prone skin, sensitivity or dry skin)
Hyaluronic acid	Synthetic detergent (syndet) ‒ Respecting the acid pH of the skin
Sodium PCA	
Ceramide	
Glycerin	
Sorbitol	
Propylene glycol

**Table 7:** Dermocosmetics (active components).

Dermocosmetics
Salicylic acid
Lipohydroxy acid
Alfa hydroxy acid
Retinol
Niacinamide
Licochalcone
L-carnitine
Decanediol
Linoleic acid
PCA zinc
Hydroquinone
Alpha arbutin
Thiamidol

### Makeup

Acne is more common in women than in men, and 58% of adult women always or almost always use makeup to improve their appearance and self-confidence.^40^ Data from the literature show that quality of life can be improved with corrective makeup. It is important to note that these studies generally involved few patients and primarily used cosmetic foundations.^41^, ^42^ Our panel of experts recommends non-comedogenic makeup for acne patients to reduce the impact of the presence of lesions at the beginning of treatment (8/8).

### Oral isotretinoin

Isotretinoin is clearly indicated for severe or refractory acne (nodular, cystic, or active scarring), but is also indicated for moderate (papulopustular) acne that does not respond to conventional therapy.^43^, ^44^

The relapse rate of patients taking isotretinoin for acne is about 30%, but it has been described as high as 50% up to five years after discontinuation of the drug, especially in severe truncal acne, younger age of drug initiation, and in patients with macrocomedos, according to various studies.^45^, ^46^, ^47^

Our group recommends that, when oral isotretinoin is required, treatment should be based on clinical evaluation and no longer just on the cumulative dose alone. If necessary, the treatment should be continued, even after 150 mg/kg (8/8). Additionally, the authors agreed that after the use of oral isotretinoin, a one-year maintenance topical treatment should be recommended for all patients in order to reduce relapse rates (7/8).^48^

Isotretinoin is a known teratogen and has the potential to cause other adverse events.^44^, ^49^, ^50^, ^51^, ^52^, ^53^, ^54^, ^55^, ^56^, ^57^ For these reasons, when isotretinoin was first released, the recommendation was that it be prescribed only by physicians (dermatologists) who were trained to manage the dosage and side effects of the drug.^58^ As time has passed, and the research pharma (Roche®), lost the patent, in many countries, the regulations have been historically relaxed, and many other medical specialties, and even primary care physicians are prescribing the drug. Our group considers that oral isotretinoin continues to be safely prescribed with informed consent signed by patients and only by physicians with experience in monitoring it (dermatologists) (8/8). This is still the rule in most developed countries and in several Latin American countries such as Brazil and Chile, and has been recommended in Colombia and Ecuador.^44^, ^48^, ^52^, ^59^, ^60^, ^61^

### Diet and nutrition

The region of Latin America and the Caribbean (LAC) is currently facing a major health problem closely linked to dietary habits, with significant economic, health, and social costs. Changes in dietary patterns have been far-reaching, with a marked increase in the consumption of unhealthy, nutrient-poor foods and sugary drinks, a shift towards eating out and snacking, and a rapid rise in the prevalence of overweight and obesity in all age groups.^62^

This particular diet, characterized by a high glycemic load and increased intake of dairy products, results in elevated levels of insulin and IGF-1, which is a type of endocrine signaling that can contribute to acne. These hormonal changes can lead to an increased rate of sebocyte proliferation, and heightened sebum production, and ultimately result in greater skin inflammation.^63^

Regarding the use of probiotics, although there are some studies evaluating the use of oral probiotics in the treatment of acne, their prescription is still not part of the routine for patients until better evidence is available (8/8).

Our group agreed that although data on diet and acne need further study, it is important to provide the most up-to-date information on the topic, such as the negative relationship with foods with a high glycemic index/load, dairy products including whey protein, and the need to eat more fruits and vegetables (8/8).

### Acne induced hyperpigmentation

Acne hyperpigmentation is a common problem. It is now known that visible light is involved in the increase of melanin, aggravating conditions such as melasma and post-inflammatory hyperpigmentation, also common in acne. Even more recent data indicate a synergistic pro-pigmentation effect between UVA and visible light, thus requiring the effective blocking of both wavelengths to prevent and optimize the treatment of these conditions.^64^

Being tropical, most Latin American countries receive large amounts of solar radiation throughout the year. This affects the treatment of hyperchromia induced by acne.^65^

Furthermore, due to migratory factors and the huge presence of miscegenation in Latin America, most patients have acne-induced hyperpigmentation and want a quick improvement (8/8).

### Acne-induced hyperpigmentation/Post-inflammatory hyperpigmentation treatment

Daily sunscreen use is also important for higher phototyped patients, especially those with Post-Inflammatory Hyperpigmentation (PIH).^66^

Regular and isolated use of sunscreen for eight weeks during the summer months resulted in improvement of PIH in African American and Hispanic women. In this study, 81% of patients noted a lightening of pre-existing hyperpigmented macules, while 59% noted a decrease in the number of macules.^67^

In the treatment of acne hyperchromia, it is important to interrupt the inflammatory process early and effectively. Whitening agents can be added to the treatment to accelerate the process. It is very important that patients are warned about the possible risks and duration of use of each type of whitening cream, especially when using hydroquinone.^68^

There are now new options with excellent safety profiles that can be used alone or in combination with other treatments, such as thiamidol-containing dermocosmetics.^69^

### Scars

#### Risk factors

One of the main goals of acne treatment is to prevent scarring. Risk factors for scarring are the severity of acne, time between onset of acne and first effective treatment, recurrent acne, and male gender.

Furthermore, a family history of acne scars, as well as the intensity of the disorder, the time without treatment, and the manipulation of the lesions influence the presence of atrophic acne scars and should be discussed with the patient prior to treatment (8/8).

A high number of patients with mild to moderate acne develop acne scarring. In a study conducted in the USA, 43% of a total of 1,972 acne patients evaluated had acne scarring, and of these, 69% had mild or moderate acne at the time of the study.^70^

Inflammation present in all stages of acne would be an important factor in their development. Therefore, early treatment should be started even in patients with mild acne to reduce the risk of scarring.^71^, ^72^

#### Early treatment

All cases of acne, even mild ones, need to be treated effectively. Scarring can occur after any intensity of acne (8/8). Topical retinoids are effective in preventing acne scarring due to their direct and indirect anti-inflammatory properties.^72^

### Procedures

There are many medical procedures available for the treatment of acne/scarring, including intense pulsed light, microdermabrasion, peels, intralesional corticosteroids, cryotherapy, and comedone extraction. For most of these procedures, the available evidence is very scarce and of very low quality. Therefore, they should be considered as adjuncts to treatment and their true benefit in acne therapy cannot be determined.^73^, ^74^, ^75^, ^76^, ^77^

Light therapies, including laser and photodynamic therapy, have shown promising results, but there is not enough evidence to recommend them for routine use. There are also no established guidelines on the optimal dose, device, timing, and frequency of use.^78^

Based on currently available data, our expert panel agreed that dermatologic procedures, such as peels and lasers, may be associated with the treatment of acne and pigmentation, but they are not mandatory and significantly increase costs for patients (8/8).

## Conclusion

These recommendations, developed by a Latin American consensus of experts, are intended to assist in decision-making in the daily practice of acne treatment and the challenges faced by dermatologists and their Latin American patients with their unique skin conditions.

The high percentage of agreement achieved in all topics, based on the available current evidence, provides support for the best acne treatment choices in both established topics and new topics that have emerged in recent years, such as social media, Generation Z, and transgender male patients.

There are some points that can still be developed/improved to help physician's daily practice, such as a quick and practical tool for evaluating and quantifying the psychological impact that acne has on patients (which is missing) as well as a greater presence of dermatologists and scientific societies, in different channels, needed to provide quality information and support decision-making.

## Limitations

The main limitation found by this expert panel was the small number of publications related to acne and its treatment, conducted exclusively to assess the locoregional characteristics and challenges in Latin America.

## Disclaimer

The recommendations in this article are based on the best available data at the time the guideline was written. The final decision to recommend a specific treatment must be made by the physician, taking into account the individual needs of the patient, as well as the disorder course.

## Financial support

Beiersdorf.

## Authors’ contributions

Marco Rocha: Conception and design of the study, data collection, analysis and interpretation of data, critical review of content, writing, critical review of the literature, final approval of the final version of the manuscript.

Franz Barnes: Critical review of the literature, writing, final approval of the final version of the manuscript.

Jemena Calderón: Critical review of the literature, writing, final approval of the final version of the manuscript.

Leonel Fierro-Arias: Critical review of the literature, writing, final approval of the final version of the manuscript.

Carlos Eduardo Montealegre Gomez: Critical review of the literature, writing, final approval of the final version of the manuscript.

Carla Munoz: Critical review of the literature, writing, final approval of the final version of the manuscript.

Obregón Jannell: Critical review of the literature, writing, final approval of the final version of the manuscript.

Patricia Troieli: Critical review of the literature, writing, final approval of the final version of the manuscript.

## Conflicts of interest

Marco Rocha: Beiersdorf/Eucerim: Speaker, consultant, researcher and advisory board member. Nature of the compensation: honorarium.

Franz Barnes: Beiesrdorf/Eucerim: Speaker, consultant, researcher and advisory board member. Nature of the compensation: honorarium.

Jemena Calderón: Beiersdorf/Eucerim: Speaker, consultant, and Researcher. Nature of the compensation: honorarium.

Leonel Fierro-Arias: Galderma México: Speaker, consultant, and Researcher. Pierre-Fabre México: Speaker and consultant. Beiersdorf/Eucerin México: Speaker, consultant, researcher and advisory board member. NAOS/Bioderma México: Consultant, and advisory board member. Nature of the compensation: honorarium.

Carlos Eduardo Montealegre Gomez: Beiesrdorf/Eucerim: Consultant. Nature of the compensation: honorarium.

Carla Munoz: Beiesrdorf/Eucerim: Speaker, consultant, and advisory board member. Nature of the compensation: honorarium.

Obregón Jannell: Beiersdorf/Eucerim: Speaker, consultant, researcher and advisory board member. Nature of the compensation: honorarium.

Patricia Troieli: Beiersdorf/Eucerim, Galderma, La-Roche Posay: Speaker, researcher, and advisory board member. Nature of the compensation: honorarium.
